# Successful Retrieval of 35 Razors From the Stomach via Upper Gastrointestinal (GI) Endoscopy: A Case Report

**DOI:** 10.7759/cureus.52856

**Published:** 2024-01-24

**Authors:** Mostafa Shehata, Hosameldin Abdelrahman Dafalla, Yashbir Singh

**Affiliations:** 1 Gastroenterology, Sheikh Shakhbout Medical City, Abu Dhabi, ARE; 2 Radiology, Mayo Clinic, Rochester, USA

**Keywords:** overture, pica syndrome, endoscopy, upper gi, sharp foreign body ingestion

## Abstract

Adult ingestion of foreign bodies in the digestive system is a common clinical challenge, often involving mentally impaired individuals, criminals, and drug dealers or occurring accidentally. Encounters with multiple sharp foreign bodies are infrequent and pose significant risks, including gastrointestinal (GI) bleeding, perforation, internal fistulas, and infection. The choice between endoscopy and emergency surgery for removal is contentious, with the less invasive endoscopy typically favored as the first line of management, depending on the foreign body's location and endoscopic accessibility. The current literature on the treatment of numerous sharp foreign bodies is sparse. This case report illustrates the successful endoscopic removal of a large quantity of sharp foreign bodies (35 half blades) from the upper GI tract, utilizing various extraction tools. It also aims to contribute to the existing literature regarding management strategies for ingested sharp foreign bodies. A comprehensive account is provided of the clinical presentation, imaging studies, consultations, and endoscopic procedures performed, culminating in the patient's safe discharge from our facility.

## Introduction

Foreign bodies in the digestive system of adults are commonly encountered, sometimes deliberately swallowed by individuals with mental health issues, criminals, or drug dealers [1], or accidentally ingested. The types of foreign bodies can vary, but it's rare to encounter multiple sharp objects [2]. Improper or delayed management of such cases can lead to serious complications, including gastrointestinal (GI) bleeding, perforation, internal fistulas, or widespread infection. There's clinical controversy over whether endoscopy or emergency surgery should be prioritized for removing multiple sharp foreign bodies [3]. However, considering that endoscopy is less invasive, it's usually the first line of management if clinically suitable, depending on the foreign body's location in the GI tract, accessibility to endoscopy, and the size and shape of the foreign body. To date, few studies have focused on treating a large number of sharp foreign bodies ingested in the GI tract.

Fortunately, most foreign bodies are expelled naturally without complications. However, intervention is necessary in 10-20% of cases due to the potential for severe complications from impaction in the GI tract. Timely detection and management are critical to preventing severe adverse events. Endoscopy is essential for managing foreign bodies in the upper GI tract [1-3]. The success rate of endoscopic removal is impressively high, exceeding 95% in most cases, attributed to the advancement of various endoscopic retrieval devices. Despite these high success rates, there are risks associated with endoscopic removal, including mucosal laceration, bleeding, infection, and even perforation [3-5]. Several factors increase the risk of adverse events following endoscopic removal, such as the patient's age, the presence of symptoms, the size and type of the foreign body, its location, and the duration of impaction. Previous studies have provided inconsistent results due to their limited scope, often including small patient groups of children and adults. Additionally, there has been a lack of comprehensive analysis considering the foreign bodies' shape and the patient's geographic location [1-2]. A recent study focused on the clinical outcomes of endoscopic removal of foreign bodies in the upper GI tract, emphasizing the shape of the ingested objects and regional differences among patients.

This is a case report of a large number of sharp foreign bodies (35 half blades) successfully removed by esophagogastroduodenoscopy (OGD) using different methods of extraction tools to achieve full management.

## Case presentation

**Figure 1:**
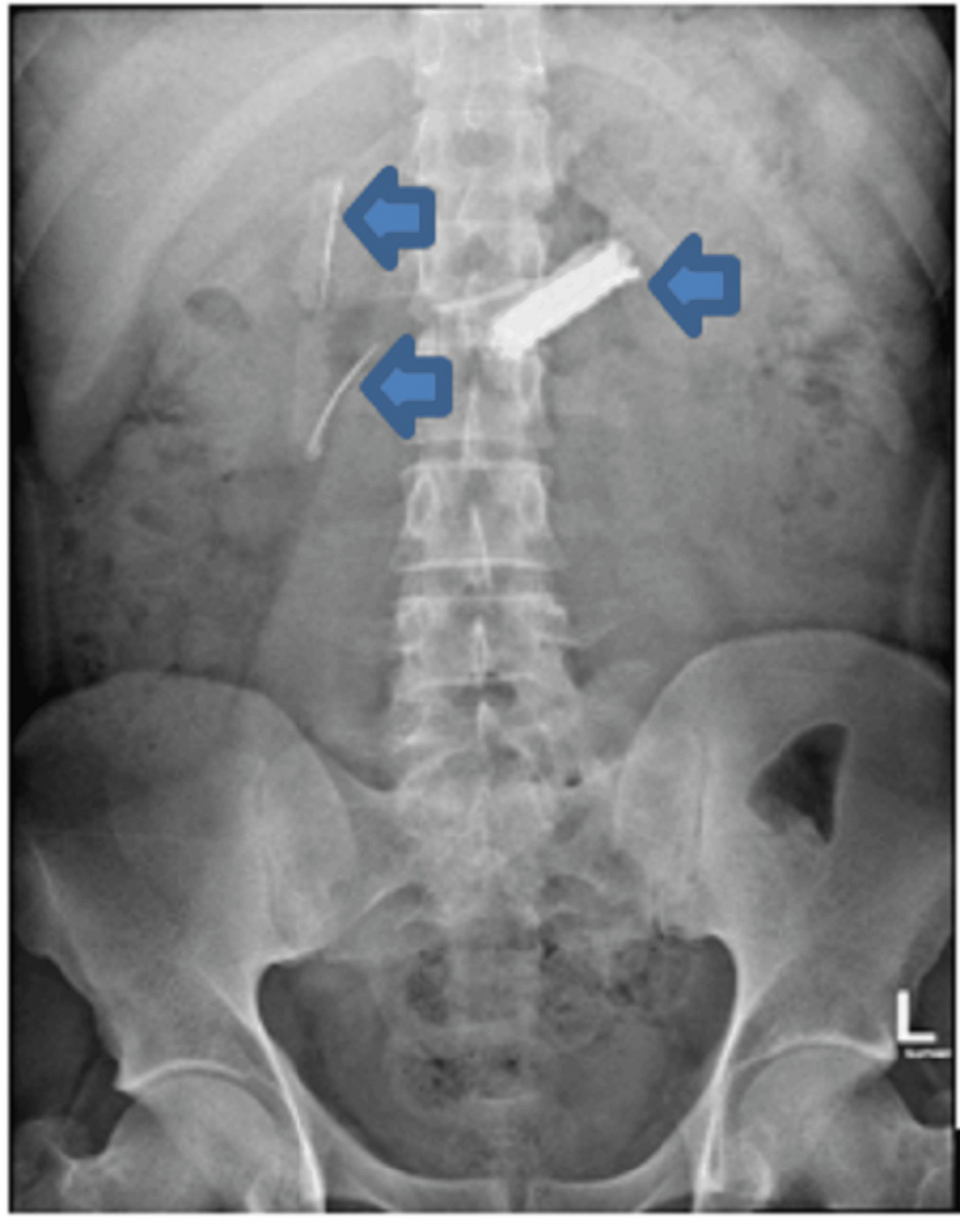
X-rays of the abdomen show sharp objects located in the stomach

**Figure 2:**
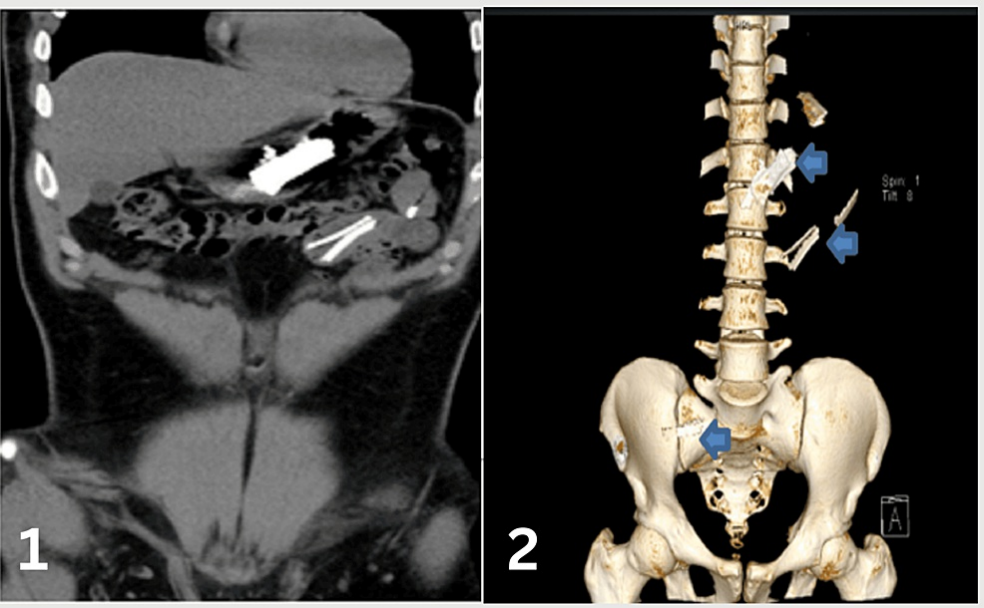
Abdomen computed tomography (CT) showed sharp objects in the stomach and small bowel (1) and a 3D bone window (2)

**Figure 3:**
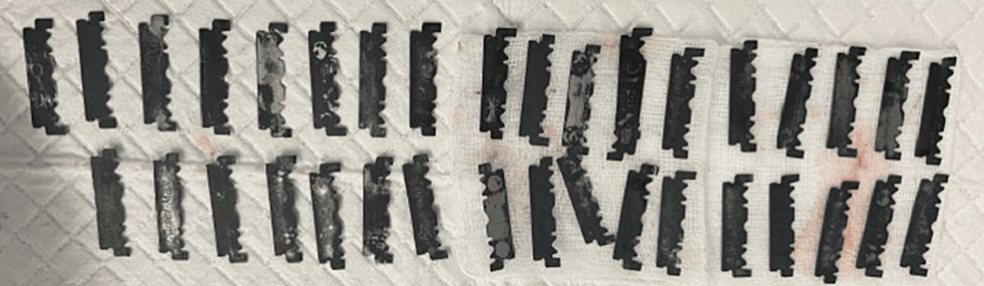
A total of 35 blades were retrieved, leaving no other blades in the stomach at the end of the procedure

**Figure 4:**
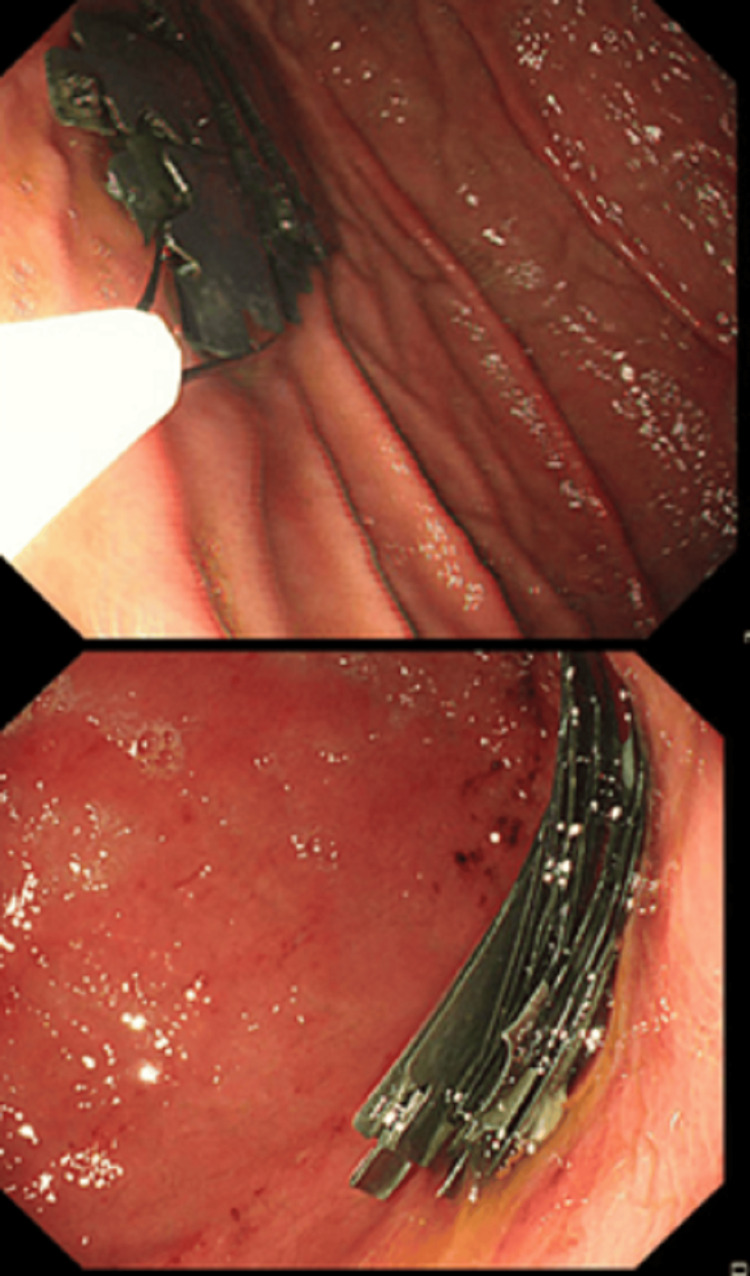
Blades retrieved from the stomach using a snare

A previously healthy 24-year-old male arrived at the hospital emergency department with a three-day history of epigastric pain. He described the pain as sharp, central, and radiating to his back, occasionally accompanied by colicky central abdominal pain. He also experienced bouts of nausea and vomiting over the past seven days. The patient denied having diarrhea or constipation and reported no symptoms related to other systems. During the examination, he was conscious, alert, and oriented, and he communicated effectively with stable vital signs. His abdomen was soft and lax with mild epigastric tenderness, and the other system examination was unremarkable. The initial workup included a complete blood count, urea and electrolytes, serum creatinine, pancreatic enzymes, and liver function tests. All results were normal, except for an elevated white blood cell count of 11.50 x 10^9/L. An abdominal X-ray revealed multiple radiopaque foreign bodies in the abdomen, located over the mid-abdomen at the level of the L1-L2 vertebral bodies, with a non-specific gas pattern and no evidence of abnormal bowel dilatation (Figure 1). The patient was referred to the gastroenterology department and admitted. A CT scan without contrast of the abdomen and pelvis showed dense, linear, sharp foreign bodies in the gastric lumen, jejunum, and proximal ileum. These were limited to the bowel lumen, with no pneumoperitoneum (Figure 2). When questioned about these findings, the patient denied ingesting any foreign bodies. Considering the sharp nature of the foreign bodies and their location within the reach of the upper GI endoscopy, and after a multidisciplinary meeting with surgery and radiology colleagues, the plan was to perform urgent OGD, which was done under general anesthesia. This procedure revealed multiple sharp razors within the stomach, and a total of 35 razors (size 4.8 x 1.2 cm) were retrieved using jumbo biopsy forceps and snares (Figure 3). An over-tube was used during the procedure to protect the esophageal wall from injury during retrieval. Most of the razors were in the fundus of the stomach and were captured by the snare or jumbo forceps and released in the antral area, then captured again in a tangential position to ensure passage through the over-tube. The endoscopy also showed linear, acute gastric ulcerations and multiple erosions (Figure 4). The procedure was performed under general anesthesia with the premedication of midazolam. The patient was intubated during the procedure to ensure airway protection and minimize any movement from the patient during the endoscopy.

**Figure 5:**
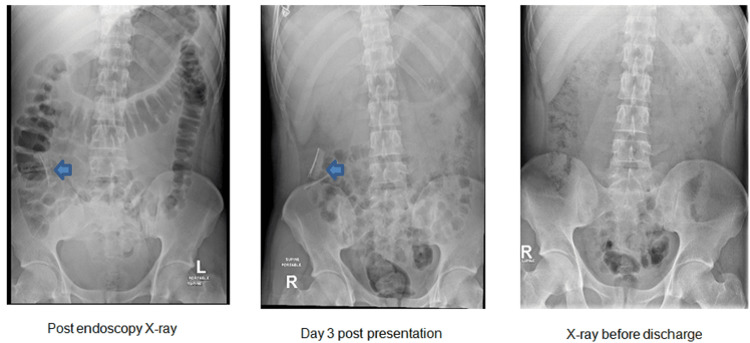
A sequence of X-ray images indicating different times during the hospital stay

The total procedure time was two hours, including endotracheal intubation time. Following the procedure, an abdominal X-ray revealed no remaining foreign bodies in the stomach, but two foreign bodies were still present in the distal small bowel, as observed in the X-ray taken upon presentation (Figure 5). The patient was moved to the ICU for observation and extubated the next day. The surgery and anesthesia team suggested keeping the patient intubated overnight, considering the possibility that the patient may need surgical intervention for the remaining three blades in the distal small bowel, to avoid re-intubation in case of need for surgery. On follow-up, there were no signs of an acute abdomen or raised inflammatory markers. A conservative management approach was chosen, involving monitoring and serial abdominal X-rays. Two days later, the patient reported the passage of metallic objects in his stool, and a subsequent X-ray showed no remaining foreign bodies within the bowel (Figure 5). The psychiatric team evaluated the patient and gave the input that this event was not suicidal and considered pica syndrome as a differential diagnosis, which is compulsive eating of non-nutritive substances and can have serious medical implications (13). Therefore, he was deemed safe for discharge from the hospital, with follow-up appointments scheduled in the psychiatry clinic.

## Discussion

The complications of ingesting sharp objects underscore the importance of timely and appropriate investigations, including X-rays and CT scans. OGD is the first line of management when the object is within the scope's reach; however, objects that are located very proximally (i.e., hypopharynx, proximal esophagus). ENT physicians can do a rigid esophagoscopy for retrieval. Upper GI endoscopy presents its own challenges and complications. During endoscopy, airway protection is crucial. Various retrieval tools, such as snares and grasper forceps, are used, along with an overture, to prevent injury to the esophageal wall [6].

In this case, we managed to retrieve 35 sharp pieces from the stomach while the remaining two blades in the small bowl passed spontaneously. This case serves as a review of the management of similar cases and highlights that endoscopic management can generally be performed safely, even with numerous sharp foreign bodies. There were also two other blades in the distal small bowel, which were observed and passed spontaneously without complication. Here, we can focus on successful endoscopic management, even with this large number of sharp foreign bodies, and the role of the gastroenterologist in exerting all efforts to remove the foreign body before considering the option of surgery. The types of foreign bodies can vary, but multiple sharp objects are uncommon [2]. The clinical debate continues over whether endoscopy or emergency surgery is more advantageous for removing multiple sharp foreign bodies [3]. Delayed or improper management can lead to severe complications, such as GI bleeding, perforation, internal fistulas, or widespread infection. The risk of perforation from sharp foreign bodies can be as high as 15% to 35% [7]. Blades, in particular, pose piercing and cutting threats to soft tissue. In this case, as the patient denied the ingestion of any foreign body, the time of ingestion is not determined; however, symptoms started 48 hours prior to the ER presentation, justifying the recommendation for timely intervention. Surgery in such cases presents challenges as comprehensive exploration often requires a large incision, which can hinder recovery. Usually, those patients have a longer hospital stay. Additionally, controlling hand strength during the surgical removal of foreign fragments is difficult, often resulting in secondary damage to the digestive tract [8]. Endoscopy is a well-established and reliable method for removing various types of upper GI foreign bodies [9]. Practice guidelines clearly state that the timing of endoscopy and the size, shape, content, and location of the ingested object(s) must be considered [10]. Extra caution is necessary when dealing with multiple sharply pointed foreign bodies due to the increased risk of perforation in certain bowel areas, such as the small bowel, ileocecal valve, and gastroesophageal junction. Using endoscopic tools like a cup and an overt tube can decrease the risk of such complications. Advancements in endoscopic equipment and the availability of various instruments, including foreign body forceps, snares, stone-capturing baskets, and balloon techniques, have helped address the challenge of removing a wide array of foreign bodies with limited types of instruments [11-12]. Although endoscopy is the preferred treatment for foreign body removal, it requires careful assessment of the patient's overall condition, similar to surgery. Laxatives or enemas for bowel preparation should be administered with caution. The case discussed involved the retrieval of a large number of sharp foreign bodies from the stomach using OGD, executed in a timely manner and following proper interpretation of the images while utilizing a multidisciplinary team model of care. This management required considerable patience and skill from both the endoscopist and the assisting technician.

## Conclusions

Based on the present case, even with the large number of sharp foreign bodies in the stomach, upper GI endoscopy is considered safe and effective for managing such cases, considering the size and shape of the foreign body and its ability to pass it through the overt tube. The case also highlights the importance of leveraging available expertise and endoscopic tools within a multidisciplinary team setting to ensure optimal patient outcomes.
